# Misconceptions about weather and seasonality must not misguide COVID-19 response

**DOI:** 10.1038/s41467-020-18150-z

**Published:** 2020-08-27

**Authors:** Colin J. Carlson, Ana C. R. Gomez, Shweta Bansal, Sadie J. Ryan

**Affiliations:** 1 Center for Global Health Science and Security, Georgetown University Medical Center, Georgetown University, Washington, DC 20007 USA; 2 Department of Ecology and Evolutionary Biology, University of California, Los Angeles, Los Angeles, CA 90024 USA; 3 Department of Biology, Georgetown University, Washington, DC 20007 USA; 4 Quantitative Disease Ecology and Conservation (QDEC) Lab, Department of Geography and the Emerging Pathogens Institute, University of Florida, Gainesville, FL 32610 USA

**Keywords:** Ecological epidemiology, SARS-CoV-2, Environmental sciences

## Abstract

Weather may marginally affect COVID-19 dynamics, but misconceptions about the way that climate and weather drive exposure and transmission have adversely shaped risk perceptions for both policymakers and citizens. Future scientific work on this politically-fraught topic needs a more careful approach.

Since the first weeks of the pandemic, substantial scientific and public attention has focused on how the weather could reduce or alter COVID-19 transmission. Whether due to increasing scientific attention on climate change and health or simply because the novel pandemic virus has forced us to look to other diseases for ideas, many were expectant—if not outright hopeful—that SARS-CoV-2 might show environmental sensitivity that curbed epidemic risk in some way. Now, clarifying and refining those expectations with available evidence is urgent, particularly in terms of how scientists communicate with policymakers and the public.

## How and why weather could affect COVID-19 transmission

A convincing argument that weather influences COVID-19 can be formulated in three parts: (1) experimental data suggest SARS-CoV-2 persistence on surfaces or in the air is sensitive to temperature, humidity, and ultraviolet light; (2) other environmentally sensitive respiratory viruses are seasonal, and more common in winter; and therefore, (3) climatic effects could be protective over space (hot, dry places might have less transmission) and time (summer might see reduced transmission compared to winter). All three are plausible and are generally consistent, but in many places (including, and especially, on social media), the basic premise of each has been communicated to the public, and policymakers, in a way that obscures key nuance and creates false confidence.

### Experimental evidence shows that SARS-CoV-2 is environmentally sensitive

Like other viruses with a lipid envelope, SARS-CoV-2 is probably sensitive to temperature, humidity, and solar radiation; this affects its ability to persist on surfaces and in air, and might have subtle impacts on transmission. But the finer details of microbiology are often lost, leading to false confidence in how lab studies could scale up to the real world. For example, studies showing that germicidal ultraviolet radiation in hospitals and laboratories (ultraviolet C (UV-C) wavelengths) kills the virus have been misconstrued as evidence that sunlight (a mix of UV-A and UV-B) would effectively neutralize the virus in outdoor public spaces, possibly at a scale detectable from case data^1^. Newer experimental evidence supports the hypothesis that sunlight might also have an effect on SARS-CoV-2^2^, although a global study found only a 1% reduction in transmission linked to environmental UV radiation^3^. In the real world, these effects will be slight, and unlikely to set hard limits on transmission anywhere in the world^4^.

### Other upper respiratory tract infections are seasonal, with declines in warmer months

Influenza, the common cold, and other respiratory infections show seasonal transmission that coincides with changes in temperature, humidity, and solar radiation. But seasonal epidemics are also a product of the transmissibility of a virus, the initial susceptibility of a population, and the degree and nature of immunity conferred by infections. In basic epidemiological models, stable “oscillations” like seasonal epidemic waves usually require some degree of immunity^5,6^; at the start of a pandemic, when transmissibility is high and immunity is low, even strong environmental drivers are unlikely to curb transmission. Previous influenza pandemics show the importance of this nuance^7^. “Seasonal” versus “pandemic” influenza refers not just to different epidemic phases, but entirely different viral strains, and population susceptibility to pandemic strains starts high enough for rapid spread regardless of the season. During the first wave of the 2009 A/H1N1 pandemic, epidemic growth was still possible in August, the most environmentally unfavorable point in the year, with immunity under 20%; months later, immunity—and consequently environmental sensitivity—may eventually have been high enough to lead to a winter-driven third wave^8^. Scientists anticipate a similar pattern for COVID-19: while the virus could develop seasonal oscillations if it becomes endemic^9^ (i.e., if pandemic control fails in the long term), current susceptibility to SARS-CoV-2 is high enough that summer weather is unlikely to be protective^10^.

### Environmental drivers could plausibly create seasonal or geographic differences in COVID-19 outbreak intensity

But those drivers’ impacts are heavily confounded by immunity, interventions, human behavior, and other details that are usually left out of models, leading to potentially spurious conclusions. For example, most available contact tracing data indicate that the proportion of indoor transmission is high^11,12^, a pattern likely caused by a combination of social contact patterns (including both number, intensity, and duration of contacts), air circulation, and potential weather drivers like sunlight or humidity. However, when studies attempt to model links between temperature and transmission, they almost always use gridded climate data or local weather data that represents the outdoors, and is unrepresentative of indoor conditions a virus particle or aerosolized cloud would actually experience in most transmission events.

Perhaps the biggest confounder, social behavior is environmentally driven and seasonal, but is rarely weighed alongside environmental and immunity drivers as a hypothesis for why infectious diseases show seasonality^13,14^. For example, school terms are seasonal and have a marked influence on social mixing patterns relevant to influenza transmission, even in pandemics^15–17^. Without individual-level transmission data, it can be difficult to distinguish direct biological impacts of weather from behaviorally mediated seasonality, and in some cases, the two are blurred (e.g., vitamin D levels are driven by both weather and seasonal behavior). Confusing the two could easily lead to spurious predictions. If behavioral patterns become unpredictable—either because of externalities like social distancing restrictions or a feedback loop between science and public risk perceptions around seasonality—attempts at forecasting the pandemic based on environmental seasonality will only become more unreliable.

## How hypotheses became policy

Despite these points of nuance, many still expect COVID-19 to show environmental sensitivity, and the topic remains a priority for research. Under normal circumstances, the work testing these ideas would happen slowly and methodically using careful “detection and attribution” methods, which identify the effects of weather on processes like disease transmission accounting for confounders, lags, and bias in climate and disease data^18–20^.

Research on COVID-19 has operated under unusual circumstances, by necessity; more scientists from varied fields are exploring case data or simulating epidemic curves than ever before. Environmental scientists joined COVID-19 research efforts early, but might have benefitted from more active collaboration with epidemiologists and virologists. For scientists not directly involved in COVID-19 response, it was often difficult to appreciate the global scale of reporting and testing bias, leading to false inferences from the best available data. In extreme cases, studies inferred broad “absence” of the virus in data-deficient countries as evidence of climatic protection, while these countries faced severe epidemics by the time the studies were published^21^.

As studies were picked up by the press and on social media, they shaped public conversations, and world leaders took notice—and as with every facet of the pandemic, basic science became heavily politicized. As of June, official UK guidance on reopening speculated that “we may well actually have some summer weather a little in our favor”^22^. In March, President Jair Bolsonaro suggested that an Italy-like crisis would be impossible in Brazil due to “climatic differences” between the countries^23^. In an April press conference, President Donald Trump observed: “Maybe this goes away with heat and light. It seems like that’s the case”^24^. Now that summer has brought the highest case totals yet in the United States, cognitive dissonance has given way to conspiracy theories, as one White House surrogate argued that the discrepancy is evidence the virus was lab-made: “Everybody thought—and this was a reasonable presumption—that come summer, the heat and humidity would get rid of the virus. It doesn’t look that way. This looks like a weaponized virus”^25^. Claims that SARS-CoV-2 is artificial or weaponized are considered false by the scientific community^26^.

Although these political interpretations are not always directly connected to scientific work, connections between individual studies and policy outcomes are surprisingly identifiable from publicly available documents. For example, a preprint from March 2020 used species distribution models to predict the full global extent of COVID-19 transmission^21^. The work concluded “a worst-case scenario of a synchronous global pandemic is improbable” and “the disease will likely marginally affect the tropics”, a claim that was soon picked up verbatim by the UN World Food Program^27^ among others. As the claim gained momentum, it was used to suggest lockdowns or other restrictions could be lifted in summer, or avoided entirely in warmer countries, in Indonesia^28^ and possibly Pakistan^29^, and continues to shape public and policy perceptions of risk (e.g., outbreak planning in Africa^30^). As the global case total passes 11 million (with 1.1 million cases in Brazil alone), the tropics have not been spared by any reasonable standard. Although the study’s predictions and design were fundamentally flawed^4^, it continues to have a lasting impact on public perceptions and policy.

## Navigating the political climate of COVID-19

While early studies found negative results and encouraged policymakers not to tailor interventions to weather^31,32^, these were largely drowned out; the “COVID-climate link” is now widely popular, independent of ongoing scientific debate. Around the world, this narrative has been used to justify avoiding lockdowns in arid countries or lifting them for the summer (although as the Southern Hemisphere enters winter, this line of reasoning has not been publicly used to advocate for reinstituting lockdown).

Uncertainty and confusion about scientific consensus sustain these narratives, sometimes producing policy outcomes that studies have explicitly warned against. When studies on the link between COVID-19 and weather are published, authors should expect to encounter a mix of good faith misunderstanding (public confusion around scientific nuance or uncertainty) and bad faith politics (economic interests driving reopening or non-intervention, or now, conspiracy theories about the virus’s origins). The signals of environmental drivers may be identifiable and interesting, but—no matter how small an effect is found, or how carefully statements are qualified—scientists who choose to produce scholarship on the topic should be prepared to have their work misread and misrepresented as indicating that some places or seasons are safe from COVID-19.

The burden of correcting misconceptions, and realigning policy, will probably fall on science communicators. It is urgent that public health guidance reiterate the best available scientific consensus: we recommend messaging focus on three key points, which synthesize most of the available evidence (Box 1). If science communicators and public health authorities reiterate these points, they could minimize future underestimation of risk.

Box 1 Summary for policymakers Weather probably influences COVID-19 transmission, but not at a scale sufficient to outweigh the effects of lockdowns or reopenings in populations. Policymakers should be aware of a few key points:

1. No human-settled area in the world is protected from COVID-19 transmission by virtue of weather, at any point in the year. Indoor transmission remains likely everywhere the virus is spreading, and outdoor transmission is still possible if other precautions (social distancing, mask use, etc.) are not taken.
2. Many scientists expect COVID-19 to become seasonal in the long term, conditional on a significant level of immunity^9^, but that condition may be unmet in some regions, depending on the success of outbreak containment. In the future, seasonality could lead to worse outcomes in the winter, but in the near term, weather is unlikely to prevent SARS-CoV-2 epidemics in the summer. Policymakers should be careful about forecasts that predict lower or no transmission in hot, dry weather.
3. All pharmaceutical and non-pharmaceutical interventions are currently believed to have a stronger impact on transmission over space and time than any environmental driver. Evidence to the contrary is currently too incomplete and disparate to change any of those interventions based on weather.
4. With current scientific data, COVID-19 interventions cannot currently be planned around seasonality. Outbreaks could easily defy expectations built on just a few months of population-level data. For example, decreased spread in the spring might lead some scientists to expect that heat directly reduces transmission, when in reality, the transmission could peak when people will aggregate indoors to escape both hot summer weather and cold winter weather. Relying on this kind of guesswork will inevitably leave policymakers unprepared.

When faced with uncertainty, rather than act based on any given scientific study, policymakers can turn to documents like the National Academies Studies on COVID-19, including their specific guidance on seasonality^7^, that synthesize and interrogate existing evidence.
